# Supplementary material for: Decomposition of the pangenome matrix reveals a structure in gene distribution in the Escherichia coli species
Source: mSphere. 2024 Dec 31;10(1):e00532-24. doi: 10.1128/msphere.00532-24 (PMC11774025; doi:10.1128/msphere.00532-24)
Supplement: Table S1 — NMF model metrics. [file msphere.00532-24-s0002.pdf]

NMF binarized reconstruction metrics (L-bin * A-bin)		
**Metric**	**Brief Description**	**Value**
Precision	How many detected signals (gene presence) are correct?	0.96
Recall	How many correct signals (gene presence) are detected? (hit rate)	0.72
F1-score	Harmonic mean of Precision & Recall	0.82
Accuracy	How many genes (presence & absence) are correctly reconstructed?	0.87

Table S1: NMF model metrics. The binarized L and A matrices are multiplied to generate a reconstruction of the P matrix. This reconstructed matrix is then compared to the original matrix and a contingency table is generated. The following metrics are calculated from this confusion matrix.
