# Supplementary material for: Decomposition of the pangenome matrix reveals a structure in gene distribution in the Escherichia coli species
Source: mSphere. 2024 Dec 31;10(1):e00532-24. doi: 10.1128/msphere.00532-24 (PMC11774025; doi:10.1128/msphere.00532-24)
Supplement: Table S2 — Specific genes found in each phylon uniquely. [file msphere.00532-24-s0003.pdf]

Phylon	# single-phylon genes	# single-phylon genes (known function)	Transcription Factors	Pili	Metabolic	Motility	Membrane- based	Phage genes	Other notable genes	Notes
**unchar-6**	11	7	0	0	4 (Fe-2+ transport, NQR complex protein, Iron permease, thioredoxin-like)	0	0	0	2 MacB-like periplasmic proteins	Transporters (potential effux pumps?)
**unchar-2**	58	24	1 (S24 peptidase-family protein)	2 (Tfp assembly protein, Tfp ATPase PulE)	2 (transport C4-dicarboxylates during anaerobic growth)	6 (Type II sec system + Tfp)	3 (dephosphorylation of heptose(II) of outer membrane LPS core, Transglycolase, conjugal DNA-protein transfer)	1 (integrase)	0	Sex pilus + F-plasmid system
**unchar-8**	40	37	3 (Fis-like protein, sigma-70 protein, 1 unknown)	0	0	37 (flagella proteins)	2 (rod-binding protein, glycerophosphotransferase)	0	0	Flagella system

**unchar-1**	26	7	0	3 (Type IV conjagative transfer system pili)	4 (Type IV secretion system, xylose isomerase-like protein, F plasmid transfer operon protein, conjugal transfer protein)		1 (transglycosylase)	0	0	Sex pilus + F-plasmid system
**unchar-5**	22	2	2 (nucleic acid-templated TF, putative anti-toxin of TA system)	0	0	0	0	2 (phage tail assembly chaperone protein, phage gp6-like head-tail connector protein)	0	Putative Phage + Toxin-Antitoxin system
**unchar-9**	15	2	0	0	1 (Phage lysozyme)	0	0	10 (Mutliple phage structure genes)	0	Phage
**unchar-4**	21	1	0	0	0	0	0	6 (putative phage genes)	0	Phage
**unchar-3**	27	2	0	0	1 (Phage lysozyme)	0	0	10 (putative	0	Phage

								phage genes)		
**unchar-7**	0	0	0	0	0	0	0	0	0	
Shigella-sonnei	48	27	1 (CP4-57 prophage regulatory protein)	0	12 (Fe-containing ROH dehydrogenase, BMCs and carboxysomes)	0	2 (periplasmic protein TonB, tail fiber protein)	2	0	Some alt carbon metabolism + BMCs/carboxysomes
Shigella-flexneri	28	12	1 (Phage terminase)	0	1 (folA-like protein)	0	0	5	2 (transposases)	Phage genes + folA-like protein
Shigella-dysentariae-boydii	13	9	1 (oxidizing proline to glutamate)	0	1 (treA-like)	0	0	0	0	treA-like gene + proline/glutamete TF
**B1-ShigaToxin**	49	24	3 (poorly characterized)	1 (part of yfcOPQRSUV fimbrial operon, increases adhesion to T24 bladder cells in absence of fim genes)	9 (PTS transporters, L-seryl-tRNA selenium transferase, folA-like protein)	2 (flagella proteins)	2 (alpha-amylase, lpxD-like protein involved in lipidA biosynthesis)	1	0	PTS transporters + yfc operon pilus (adheres better to bladder cells)

**B1-ST678**	54	18	6 (Peptidase S24-like, HTH lactose operon repressor, cold-shock-like protein, AMP salvage TF, antitoxin of ParD family)	0	2 (PEP-dependent PTS enzyme IIA & lactose/cellobiose-specific IIB subunit)	0	2 (Secretion protein, Enterobacterial Ail/Lom protein)	1	0	lactose/cellobiose-specific PTS, lactose operon repressor, cold-shock-like protein, AMP salvage TF
**B1-other**	1	0	0	0	0	0	0	0	0	1 uncharacterized gene
**C**	18	9	0	1 (fimbrial protein)	3 (Glycosyl hydrolase, alpha-amylase, hyrdogenase)	0	1 (glycosyl transferase)	1	2 (recombinase, protease)	Fimbrial protein + glycosyl hydrolase/transferase, known QREC
**A-Thailand**	3	2	0	0	0	0	0	0		1 putative VF
**A-hot**	9	4	1 (Antirepressor)	1 (pili assembly protein)	0	1 (tail fiber assembly protein)	0	0	1 (SpoVT AbrB-like protein)	Pilus genes + Toxin-Antitoxin system +

										Antirepressor
**A-BL21**	10	3	1 (Bacteriophage CI repressor)	0	0	0	1 (autotransporter)	2 (Bacteriophage repressor, putative Caudovirales tail fibre assembly protein, lambda gp2)	0	Phage genes
**A-K12**	126	64	13 (yfdM, dicA, yedL (Nitrogen, NtrC-2, NtrC-3), yagJ (CP-46 prophage) , dicC (Qin prophage) , xynR (CP-46 prophage) , ynaK (Rac prophage) , appY (LPS, ArcA), yfdO	0	19 (cytochrome b562, thiamine pyrophosphate enzyme, yagF, yfjY (CP4-57), transmembrane transporter, ilvB, PTS sorbose-specific IIc component, glvC, nagA, rfbC,	3 (2 outer membrane proteins, 1 flagellin protein)	11 (wbbK, stfR (Rac), ompN, putative puromycin resistance, rhsD, stfQ (Qin), wbbI, yaiT and other putative transporters)	3 (intF integrase, rnlA (CP4-57), ynaA (Rac))	0	wbbK and wbbI, CP4-56, CP4-57, Rac, and Qin prophage genes, yedL

			(CPS-53 (KpLE1) prophage) , perR)		yagG, yagH, glvB, yagE, various transporters)					
**A-main**	0	0	0	0	0		0	0	0	
**A-Sanji**	25	9	1 (Phage terminase)	0	1 (gnd enzyme (PPP))	0	4 (3 Glycosyl transferases group 1 proteins, 1 sugar export protein)	5 (putative HK97 (-gp10) phage proteins)	0	glycosyl transfersaes + phage genes
**E-ST11**	416	200	12 (alpA (CP4-57 regulatory protein), HOCl (hypochlorite) stress regulator, and various poorly characterized TFs)	14 (yraHIJK operon genes (T24 bladder), yfcOPQRSUV operon genes (T24 bladder), elfADCG-ycbUVF operon genes (abiotic	54	12 (various flagella proteins)	24 (papD, UidB (GusB) glucuronide transporter helper protein, UDP-Ara4O to UDP-L-Ara4N, various transporters)	23 (lambda phage genes)	17 (1 acc amr gene: Ecoli_C91661 (catB3, phenicol antibiotic), 16 secretory pathway proteins)	yra, yfc, elf-ycb operon pili, papD-like, catB3 (AMR)

				surfaces))						
**D-ST32**	33	6	1 (CP4-57 regulatory protein AlpA)	0	0	0	0	4	0	Phage + many unchar genes
**D-ST405**	31	9	2 (dicB repressor, ParB-like protein)	0	0	1 (poorly characterized)	1 (cell adhesion protein)	4	0	cell division related TFs + Phages
**F**	2	2	0	0	0	0	0	0	2 (transposes)	2 Transposases
**D-ST38**	27	18	2 (poorly characterized)	0	10 (ethanolamine utilization genes, formate C-acetyltrasferase, BMC proteins, alcohol dehydrogenase, AdoMet formation enzyme)	0	1 (Opacity family porin protein)	0	0	2 TFs, ethanolamine + BMC utilization
**D-ST69**	17	5	1 (poorly characterized)	0	0	1 (Type VI secretion protein DotU)	1 (ImpA domain protein)	1 (phage late control gene D	0	1 TF, 1 secretory protein DotU, 1

								protein)		phage protein
**G**	12	0	0	0	0	0	1 (outer membrane heme hemoglobin receptor)	2 (Bacteriophage replication protein O, Inovirus Gp2)	0	Outer membrane proteins (potential OmpT-like gene)
**B2-main**	23	13	1 (regulates arginine biosysnthesis)	3 (2 fimbriae, 1 yfcOPQRSUV operon gene (T4 bladder))	7 (Amidinotransferase, carbamate kinase family protein, tabA - influences biofilm formation by repressing fimbria in 8 hours, L-citrulline biosynthessis protein, glpX, glycerol catbolism)	0	2 (cell adhesion, autotrasporter)	1	0	Arginine, L-citrulline, -> possible nitric oxide production ?

**B2-ST131**	124	54	9 (Peptidase S24-like, lacI family TF, IclR HTH, sucrose operon repressor, repressor controlling sicalic acid biosynthesis, other unchar TFs)	0	19 (pyruvate formation via Schiff base, sterol desaturate, PTS-trehalose-specific, Malonate decarboxylase transporter, invertase, other poorly characterized proteins)	1 (Mediates taxis to ribose & galactose via interaction with periplasmic ribose- or galactose-binding proteins)	8 (various transporters including for sicalic acids)	18 (FluMu proteins)		0	Sugar transporters and metabolism genes including sialic acids

Table S2: A list of specific genes found in each phylon uniquely. The number of single-phylon genes (genes found only in strains of 1 phylon and not any others) is listed. Gene classifications of interest are also listed (pili, transcription factors, etc).
